# Counseling Customers: Emerging Roles for Genetic Counselors in the Direct-to-Consumer Genetic Testing Market

**DOI:** 10.1007/s10897-012-9548-0

**Published:** 2013-04-01

**Authors:** Anna Harris, Susan E. Kelly, Sally Wyatt

**Affiliations:** 1 ESRC Centre for Genomics in Society, Egenis, University of Exeter, Byrne House, St German’s Road, Exeter, EX1 4PJ UK; 2 Technology & Society Studies Department, Faculty of Arts and Social Sciences, Maastricht University, PO Box 616, 6200 MD Maastricht, The Netherlands

**Keywords:** Genetic counseling, Internet, Direct-to-consumer genetic testing, Discourse analysis

## Abstract

Individuals now have access to an increasing number of internet resources offering personal genomics services. As the direct-to-consumer genetic testing (DTC GT) industry expands, critics have called for pre- and post-test genetic counseling to be included with the product. Several genetic testing companies offer genetic counseling. There has been no examination to date of this service provision, whether it meets critics’ concerns and implications it may have for the genetic counseling profession. Considering the increasing relevance of genetics in healthcare, the complexity of genetic information provided by DTC GT, the mediating role of the internet in counseling, and potential conflicts of interest, this is a topic which deserves further attention. In this paper we offer a discourse analysis of ways in which genetic counseling is represented on DTC GT websites, blogs and other online material. This analysis identified four types of genetic counseling represented on the websites: the integrated counseling product; discretionary counseling; independent counseling; and product advice. Genetic counselors are represented as having the following roles: genetics educator; mediator; lifestyle advisor; risk interpreter; and entrepreneur. We conclude that genetic counseling as represented on DTC GT websites demonstrates shifting professional roles and forms of expertise in genetic counseling. Genetic counselors are also playing an important part in how the genetic testing market is taking shape. Our analysis offers important and timely insights into recent developments in the genetic counseling profession, which have relevance for practitioners, researchers and policy makers concerned with the evolving field of personal genomics.

## Introduction

While genetic testing has been an established clinical practice for some time (de Vries 2008), it has only been in recent years that direct-to-consumer genetic testing (DTC GT) has emerged as part of what is now a thriving healthcare marketplace. Through the internet, people currently have access to a variety of genetic testing products which make claims about establishing paternity, ancestry and predisposition to a growing number of traits, diseases and conditions.

The prevalence of genetic testing products being advertised and sold on the internet has been a source of concern for healthcare providers and professional organizations, governments and other regulatory and consumer groups. One common point of concern is how individuals may (mis)understand, (mis)interpret and cope with the genetic information that they are provided from genetic tests not ordered through a healthcare professional (American College of Obstetricians and Gynecologists 2008; Couzin 2008, p275; Hudson et al. 2007; National Society of Genetic Counselors 2010a). More specifically, DTC GT companies have been criticized for providing genetic information outside the context of genetic counseling (Hennen et al. 2010; Nuffield Council on Bioethics 2010; Udesky 2010; Wade and Wilfond 2006, p285).

This critique has been accompanied by calls for the provision of pre- and post-test counseling within the industry (American College of Medical Genetics Board of Directors 2004; American College of Obstetricians and Gynecologists 2008; Jordens et al. 2009). In this paper we examine the provision of genetic counseling in the emerging DTC GT market, analyzing how these calls for genetic counselors’ involvement in the industry are being met.

There is now an increasing body of literature, principally from the disciplines of ethics and sociology, beginning to address the social, ethical and political aspects of DTC GT (e.g. Borry and Howard 2008; Nordgren and Juengst 2009; Parthasarathy 2010; Williams-Jones and Graham 2003). In general, much of this research assumes a shared understanding of DTC GT as the selling of genetic tests to the public unmediated by a physician (e.g. Hennen et al. 2010, p170; Hock et al. 2011, p325; McGowan et al. 2010, p265; Richards 2010, p293). In this paper we define DTC GT as the offer (advertising or selling) of genetic testing services to consumers, whom we define as the general public, patients *and* physicians. We include physicians in our definition of consumers as many of the genetic testing products are marketed either directly to doctors, or to doctors and the public/patients simultaneously. The involvement of doctors as consumers of DTC GT is heterogeneous, including those who directly order the test and those who sign forms supplied by their patients. Others have recognized the trend towards advertisement of DTC GT to doctors, and noted that this, as well as the involvement of in-house physicians within these companies, is an ethically problematic development in this industry (Howard and Borry 2011).

This broader definition of consumer is important in considering the evolving role of physicians in the DTC GT process, and we discuss this issue further in relation to physicians’ engagement with genetic counselors employed within the industry. It is also worth noting here that our definition of DTC GT encompasses a wide variety of commercial enterprises, including well-established genetic testing services that specialize in monogenetic and rare genetic diseases and newer companies offering susceptibility testing for a large number of common complex health conditions alongside traits and other non-health related information.

Despite the calls for genetic counseling to be provided with DTC genetic tests, concerns have been raised about how and with what implications such counseling would be provided. Concerns have included how consumers will be influenced in their beliefs about genetic testing by advertisements and arguably exaggerated (Hock et al. 2011, p325) claims found on the DTC GT websites (Wade and Wilfond 2006, p288).

Concerns have also been raised about genetic counselors making preventive health recommendations, such as behavior change, from DTC GT results. These results are often based on genomewide association study (GWAS) data for common conditions which provide relatively small contributions to predictions of disease risk (O’Daniel 2010). Health behavior recommendations from these predictions are often similar to those for healthy living in general (Leighton et al. 2012, p20).

It has also been suggested that preventive health advice in this context sits problematically with non-directive genetic counseling (Rees et al. 2006), a practice that is being reframed in the context of health promoting medical settings (Koch and Nordahl Svendsen 2005). There is a general concern about limited family history taking, and more broadly, whether DTC GT falls within genetic counselors’ scope of practice (Clarke and Thirlaway 2011; Hock et al. 2011). Conversely, some have argued that the genetic counseling profession needs to embrace the developments occurring in DTC GT, recommending that counselors use their genetic expertise productively in order to provide service to DTC GT customers and their healthcare providers (O’Daniel 2010; Weaver and Pollin 2012).

The provision of genetic counseling within the DTC GT industry is thus emerging as a controversial issue and can be seen to be challenging many of the “traditional” roles of counselors. Following the National Society of Genetic Counselors (NSGC) (2011) and the American Board of Genetic Counseling (ABGC) (2009), we define genetic counseling as providing information and support to individuals and families at risk of genetic disease. We use the definition provided by these American organizations considering that most websites we analyzed were registered in the U.S. According to the NSGC and ABGC, counseling includes interpretation of family, medical and psychosocial histories in order to assess genetic risk, as well as the provision of modified information, in response to verbal and nonverbal cues, in a “culturally responsive” and “non-coercive” manner (American Board of Genetic Counseling 2009, p2) that promotes decision making and guides prevention and disease management.

Although we recognize that the distinction between “traditional” and “non-traditional” genetic counseling is now blurred (Finucane 2012, p3), central tenets of genetic counseling are missing from the DTC GT context, such as strong interpersonal skills and emotional intelligence through face-to-face counseling (Finucane 2012, p5), with time provided to explore patient values and life experiences, where knowledge about the individual and family is brought into conjunction with pedigrees and other shards of information collected through the counseling encounter (Featherstone et al. 2006, p39).

Professional roles and boundaries are often disrupted by technological change, as Petrakaki et al. (2011) highlight in their study of how communication technology is shaping the profession of community pharmacists in England. Although most genetic counselors work in clinical or hospital settings (National Society of Genetic Counselors 2011), genetic counselors have also been working in the commercial context for some time, such as in pharmaceutical, insurance and biotech companies. [The NSGC (2010b) Professional Status Survey states that 9 % of genetic counselors work in commercial laboratories and 20 % work in settings other than non-clinical settings, including commercial industry]. DTC GT presents a new context for genetic counseling however, and the diversity of opinions mentioned above reflects the importance of this industry in potentially shifting roles within the profession.

The history of genetic counseling has been continually shaped by technological advances (Kenen 1997; Pagon 2002), and the profession has undergone previous shifts in terms of roles and boundaries (Novas and Rose 2000, p493). In the 1950s the profession started to move from a strategy of public education towards an emphasis on non-directive counseling, in order to distance counseling from prior eugenic practices. In the 1970s, genetic counseling became more focused on the communication of genetic risk, with another more recent shift taking place due to the pre-symptomatic testing of the self-directed, self-responsible client (Novas and Rose 2000). Kenen (1997) documents shifts that occurred in the American genetic counseling profession in the 1990s, in part as a result of healthcare restructuring according to principles of managed care. It has been suggested that the increasing emphasis in clinical settings on genetic predisposition to common complex diseases is instituting another professional shift (Pagon 2002). We situate DTC GT within this historical context, as a technology engendering another important shift in the continually evolving genetic counseling profession, prompting reflection upon what it means to be a genetic counselor in this “genomic era” (Guttmacher and Collins 2003).

Considering the emphasis of many European and North American medical organizations on the need for genetic counseling in DTC GT, there has been surprisingly little empirical investigation of the representations of genetic counseling in the DTC GT industry. Hennen et al. (2010), and authors of a recent publication from the Genetics and Public Policy Center (2011) document the presence of genetic counseling provided by DTC GT companies, but provide little analysis. In the small amount of genetic counseling literature concerning DTC GT (e.g. O’Daniel 2010), there has been little acknowledgement of genetic counselors’ employment by DTC GT companies, a silence which we may read as “boundary work” or demarcation (Nancarrow and Borthwick 2005). This is surprising given the concerns that many medical organizations have expressed about potential conflicts that may arise when genetic counselors are employed by the genetic testing company (American College of Obstetricians and Gynecologists 2008; European Society of Human Genetics 2010; Genetics and Public Policy 2006).

In this paper we examine the role genetic counseling is coming to play in the DTC GT industry. In particular we are interested in how genetic counseling is *represented* by the DTC GT websites, examining the types of genetic testing products with which counseling is offered, the forms of counseling offered and the roles attributed to counselors. Our study does not profess to make any claims about how genetic counseling sessions are conducted in the DTC GT setting, but rather examines the *representation* of genetic counseling online, which is important when considering how this market is taking shape and the potential directions in practice. We analyze how the critiques of the lack of involvement of genetic counselors in DTC GT are being played out online.

More specifically, we critically explore how genetic counseling expertise is represented on these websites, as well as in other online material such as blogs. We argue that the DTC GT industry is potentially resulting in a change in how genetic counselors’ professional roles are being represented and consider how these emerging roles sit with more “traditional” aspects of genetic counseling. We also argue that genetic counselors have an important part to play in how the DTC GT market is taking shape, particularly in terms of the mediation of testing, counselors’ engagement with physicians, and the entrepreneurial activities of some genetic counselors, as many of these practices are moving DTC GT more firmly toward medical products and information.

## Methods

### Sample and Procedures

In December 2010, a list of websites for companies offering genetic testing services to consumers was compiled by the first author after consulting recently published literature (e.g. Hennen et al. 2010), and a list of companies compiled by The Genetics and Public Policy Center (2010). This was supplemented with online searches using various search engines (Google, Yahoo, Bing and Ask)—by entering terms such as “genetic testing” and “direct to consumer genetic test”—and TouchGraph, a weblink visualization tool—by typing in the term “genetic testing.” This generated a list of 52 websites in total. In January 2011, the first author analyzed the content of these websites to delineate which companies explicitly offered genetic testing for mental health conditions as this was the initial focus of our research. Twenty companies were identified as meeting these criteria, which offered a diverse range of genetic testing services. Although the focus on companies selling tests for mental health conditions has had an influence on the kinds of companies in our cohort, the 20 companies in our list included the most prominent, discussed and utilized companies in the market. These sites were then further analyzed to obtain a list of those companies which also offered genetic counseling. The sites were entered into the Wayback Machine program, a website which provides archived webpages, to find previous versions of websites for each company in order to examine the history of counseling provision within the companies. In searching the internet to find companies selling genetic tests, other websites were also identified which related to DTC GT and genetic counseling. A Google search was also performed to find fora, blogs and other sites where DTC GT and genetic counseling were discussed. Our intent with this methodological approach was to obtain as full a picture as possible of the online representations of genetic counseling in association with DTC GT. Because the research did not include human subjects, the local ethics committee (Institutional Review Board) confirmed that the research was exempt from review.

Data collection yielded six DTC GT websites offering genetic counseling either internally (by company employees) or by a third party; three blogs administered by DTC GT companies which discussed genetic counseling services; four blogs discussing genetic counseling and DTC GT, which were not administered by DTC GT companies; and one interview with a genetic counselor which discussed DTC GT.

### Data Analysis

We archived all web material using WebCite and transferred written text to tables for ease of analysis. The first author performed discourse analysis of all material. Discourses were considered online texts in the form of the words, images and hyperlinks on company websites, whose combined meaning extends beyond the text itself (Denzin and Lincoln 2011). We were concerned with the companies’ accounts of genetic counseling practice and thus studied representations of genetic counselors’ roles within the industry. Discourse analysis drew upon a social constructionist epistemology which recognizes that knowledge is situated and that language and discourse have a role in the way in which the genetic counseling profession is conceptualized (Hall 1997). This strategy was appropriate considering our interest in how genetic counseling is represented in discourses online, recognizing that our interpretation of these texts is only one of a number of possible readings. Analysis involved detailed and repeated readings of the texts, including visual material, looking for themes (Lupton 1997, p108). All three authors discussed these themes and further developed the analysis. We also analyzed seven position statements by healthcare organizations in relation to direct-to-consumer genetic testing and performed a literature review of research related to the DTC GT industry and genetic counseling, genetic counseling more broadly and telegenetics.

## Results

Of the 20 companies we identified, 14 did not provide genetic counseling (although several, such as EasyDNA, suggested that customers pursue genetic counseling), five did provide counseling (DeCODEme, GeneDx, Lineagen, Navigenics and Pathway Genomics) and one company offered what we have referred to as “independent” counseling (23andMe) (see Table 1). Several of these companies claimed to be the only genetic testing company to offer genetic counseling as part of the testing service. Our analysis found that two of these companies had been providing counseling services for over 10 years, whereas two companies started offering counseling in 2008. The websites varied in how they presented their genetic counseling services, with some listing the service in FAQs, and others promoting counseling prominently under “what we offer” or “about us.” All counselors represented on these websites were women, except for two men employed at GeneDx. Genetic counseling was available by phone, Skype or email—what some researchers refer to as “telegenetics” (O'Daniel 2010)—with three companies offering the counseling pre- and post-test, and three post-test only. Of the companies offering genetic counseling, all websites were registered in the U.S. except for DeCODEme.

**Table 1:** Direct-to-consumer genetic testing companies offering testing for mental illness and genetic counseling services

Company	Psychiatric genetic test offered	Target audience (in the words of the company)	Type of genetic counseling service offered	How is counseling offered? (initial contact/service provision)	Other forms of genetic interpretation provided on website
23andMe	Antidepressant response, heroin addiction, Naltrexone treatment response, nicotine dependence, alcohol dependence, ADHD, bipolar disorder, OCD, Tourette’s syndrome, schizophrenia.	Customers.	Independent: the genetic counseling is offered by an external company at additional cost, by counselors trained in 23andMe interpretation techniques; customer’s choice to take up counseling.	(via InformedDNA) Email and phone/phone and “web technology.”	A detailed report is sent to customer. There is also a community forum for customers to discuss results.
Pathway Genomics	Alzheimer’s disease and drug sensitivity.	Customers and physicians.^a^	Discretionary: the counselor is one of their experts; included as part of the advertised service.	Online request form/email (pre- and post-test).	An “easy to understand report” is posted online for customer to access.
GeneDx	Autism.	Patients including families (predominantly with rare genetic conditions).^b^	Integrated: counseling is always part of product for prenatal testing and integral to other testing.	Email and phone/unspecified.	No other forms of interpretation offered. Results are sent to the patients’ physician.
Lineagen	Autism.	Primarily physicians, also families.	Discretionary: the genetic counselor has a role as physician educator; included as part of advertised service.	Phone/phone and email (pre- and post-test).	Results are provided in a binder with “easy-to-read in formation for physicians and families” with sections including Test Results, Next Steps, and Counseling
DeCODEme	Alzheimer’s disease.	Customers.	Discretionary: not a prominent part of their service, and offered at extra cost by a third party for customers in some U.S. states.	(via InformedDNA in US) Phone/phone and email.	The customer is provided with a summary report “with a concise description of your genetic risk.” There are also links to a community forum and a video.
Navigenics	Alzheimer’s disease and drug sensitivity.	Physicians.	Discretionary: the genetic counselor has a role as physician educator and as a mediator between physician and customer; included as part of advertised service.	Phone/phone and email. Face-to-face counseling is outsourced.	Interested customers are emailed a “comprehensive guide” that provides information on genetics and genetic analysis services. There are also information sheets for doctors.

^a^Company states that they are not DTC as physicians or genetic counselors order the test

^b^Company states that they are not DTC as physicians or genetic counselors order the test

The details of these companies are provided in Table 1. Some companies that we studied did not consider themselves as “direct-to-consumer” because a physician was required to order the test. We have included these companies in our group nonetheless, because as discussed earlier, we include physicians in our definition of consumers of DTC GT. Three of the DTC GT companies administered blogs which we also analyzed wherever they discussed genetic counseling: the Navigenics Blog, The Spittoon (23andMe) and the Pathway Genomics blog. We also analyzed four blogs which were not administered by DTC GT companies: DNA Exchange, Wellsphere, PsychCentral and the Western States Genetics blog.

### Types of Genetic Counseling

From our analysis of this online material we identified four representations of genetic counseling provision in the market: the integrated counseling product, discretionary counseling, independent counseling, and product advice. Each of these is described below. (In this section, all quotations are from analyzed web material. Internet addresses are provided in Table 2):

**Table 2:** List of websites used in analysis

*23andMe*: https://doi.org/www.23andme.com
*DeCODEme*: https://doi.org/www.decodeme.com
*DNA Exchange*: https://doi.org/thednaexchange.com
*GeneDx*: https://doi.org/www.genedx.com
*Lineagen*: https://doi.org/www.lineagen.com
*Navigenics*: https://doi.org/www.navigenics.com
*Navigenics blog*: https://doi.org/www.blog.navigenics.com
*Pathway Genomics*: https://doi.org/www.pathway.com
*Pathway Genomics blog*: https://doi.org/blog.pathway.com
*PsychCentral*: https://doi.org/psychcentral.com/blog
*The Spittoon*: https://doi.org/spittoon.23andme.com
*Wayback Machine:* https://doi.org/web.archive.org
*Wellsphere*: https://doi.org/www.wellsphere.com
*Western States Genetics blog*: https://doi.org/www.westernstatesgenetics.org/wordpress

- *Integrated counseling*: the genetic counseling service was marketed as an integral part of the genetic testing product, as in the case of GeneDx. GeneDx had been providing genetic counseling for over 10 years, as part of their genetic testing service which specializes in rare hereditary disorders. This model of genetic counseling provision closely resembles the more “traditional” role of genetic counseling. By being integral to the testing product, genetic counseling is not provided at the discretion of the consumer. Rather, determination of test appropriateness, and personal interpretation, are represented as part of the testing process, following a clinical services model.
- *Discretionary counseling*: the consumer chooses whether to contact the DTC GT company’s genetic counselor, for counseling about their own or their patients’ test results. Companies vary in how prominently they advertised this service: Navigenics claims that “genetic counseling from a qualified professional is a critical part of the genetic testing experience,” and Pathway Genomics advertises counselors in their panel of “our experts,” while DeCODEme mentions genetic counseling only briefly in their FAQ section and in sample reports.
- *Independent counseling*: a service that is offered by an external company. In our group of sites, two companies - 23andMe and DeCODEme - offered genetic counseling in this way, both through InformedDNA. Independent counseling is a sub-type of discretionary counseling, where the consumer chooses to access the service. Independent counseling is consonant with 23andMe and DeCODEme’s empowerment framework that emphasizes individual genetic information as a right and consumer choice. 23andMe states that they deliberately “engaged” InformedDNA genetic counselors who are “trained in 23andMe’s unique reports and processes,” in order to “ensure that the information our customers receive is completely objective.” In their blog, The Spittoon, a post declares that by doing this the company is adhering to European Society of Human Genetics recommendations that counseling is provided independently of the company.
- *Product advice*: a form of genetic counseling that concerns information provision about the test for sale, which in some circumstances can be seen to be very similar to traditional pre-test counseling. For example, on the Navigenics blog, genetic counselors are described as helping in the decision about whether testing is appropriate. Product advice may also be given to physicians. One GeneDx genetic counselor saw herself as playing a vital role in keeping clinics up to date on test offerings.

### Genetic Counseling Roles

Interrelated with various types of genetic counseling provided by the DTC GT companies is the representation of the roles of genetic counselors. In general, genetic counselors are represented on the DTC GT websites as personal genetics experts. They are represented as being constantly “on-call,” with websites advertising “unlimited access,” counselors’ availability “7 days a week,” where they are ready “to help you at any time.” The counselors’ professional knowledge is emphasized through markers such as board certification, ethical guidelines, hyperlinks to well-respected websites such as the National Society of Genetic Counselors and scientific research. The genetic counselors’ expertise is evident in various, overlapping roles as: genetics educator; mediator; lifestyle/health advisor; risk interpreter; and entrepreneur.

- *The genetics educator*: the genetic counselor is represented on a number of sites as having a role in the education of the general (pre-symptomatic, genetically curious) consumer about genetics and the genetic testing product, through the provision of web-based information or pre-test counseling. Genetic counselors are also represented as having an important role in the education of healthcare providers about genetic testing. As examples of the latter, Lineagen offers genetic counseling services primarily as an informational service for physicians. Navigenics genetic counselors are defined as “trained healthcare professionals that specialize in personal genomics,” who are “dedicated to providing you with the most accurate information to help you, your family, and even your doctor.” Pathway Genomics genetic counselors are there for when “a physician or staff member is in need of assistance with clinical information, report interpretation or general customer service.” One genetic counselor regards this as a shift into physician education, asking on the DNA Exchange blog, “will we move from being educators of patients to being educators of health professionals?”
- *The mediator*: in many ways part of their educational role, the genetic counselor on some of the analyzed sites also acts as a mediator between the customer and the physician. A good example can be found in the genetic counseling service offered by Navigenics, where their counselors can help the customer “determine what to focus on with your doctor—or even speak to your doctor directly if he or she has questions.” The genetic counselor can also be viewed as a mediator between the customer and the DTC GT company, through their online or phone interactions.
- *The lifestyle/health advisor*: the genetic counselor offers advice about lifestyle and health behavior change about a range of complex and rare illnesses as a result of the DTC GT results. Lineagen claims that they offer “counseling to promote informed choices and adaptation to the risk or condition,” and that their counselors help families to understand “what may happen in the future and what treatment or management options are available … (and make) adjustments to the condition and choose courses of action that are best for the children and their families.” Navigenics helps the consumers to integrate information from their test results into their lives, and DeCODEme offers “counseling to help patients understand what their test results mean for their future health.”
- *The risk interpreter*: the genetic counselor is represented on websites as a specialist, an expert in risk interpretation. In general, risk interpretation is based principally on the consumers’ genomic information obtained from the genetic test. There was no mention of the potentially unspecific or uncertain nature of information provided by DTC GT on the websites we analyzed or of the difficulties interpreting genetic information without the context of a detailed family history.
- *The entrepreneur*: a number of genetic counselors have founded websites which provide telegenetics services, and have been advocates for the important role of genetic counseling in DTC GT. For example, Jordanna Joaquina is a genetic counselor and founder of AccessDNA (now called Inherited Health, which was recently bought by InformedDNA), then “a leading online consumer resource for genetics,” which educated and directed people towards testing services and counseling services. In a guest post for the DNA Exchange blog, Joaquina states that “in this brave new world of personalized medicine, I imagine that every person will have their own personal genetic counselor.”

Genetic counselors were represented on the websites and blogs we analyzed as practicing in some or a combination of these roles. The kinds of roles for genetic counselors on each website generally aligned with the company’s targeted consumer, whether this was a proactive customer, patient, carrier^1^ or healthcare provider.

## Discussion

In our analysis, we have identified various forms of genetic counseling represented on the websites, many of which are emerging as a result of genetic counselors’ involvement in the direct-to-consumer genetic testing industry. Changes in professional roles are taking place within a broader context of ongoing changes in genetic testing processes, genetic research and genetic result interpretation. The commercialization of counseling on the internet and the increasing complexity of the genetic information these internet counselors are dealing with, are contributing to shifts in the profession.

Whilst the DTC GT industry is extremely heterogeneous, we can nonetheless make some general claims about how genetic counseling is represented in the direct-to-consumer genetic testing field. First, the internet provides various forms of mediation that may be shifting boundaries of the profession, its practice and expertise. The genetic counselors on the sites we analyzed contacted individual consumers either through email or phone. Unlike the use of the telephone and internet to reach remote or hard to reach patients—how telegenetics is most commonly used (Abrams and Geier 2006; Stalker et al. 2006; Zilliacus et al. 2010)—the service is potentially available for all DTC GT consumers, regardless of their geographic location or ability to attend face-to-face sessions. Mediation of the counseling session through telephone and email also challenges genetic counselors’ abilities to read non-verbal cues, and to engage in emotive, embodied interaction (Arribas-Ayllon et al. 2012), often in a relational context with other family members (Hawkins and Ho 2012). Various internet platforms also meant that genetic counseling provision and expertise sat alongside other forms of genetic interpretation such as information sheets and online fora. These alternative forms of interpretation can be used by consumers to find out risk information, discuss the implications of their results and find further resources. Most importantly for this analysis, genetic counselors’ roles were represented as changing shape, with shifting forms of expertise promoted on the DTC GT websites. These roles include ways in which counselors educate physicians and provide product and lifestyle advice, both of which we explore in more detail below.

### Physician Education

While genetic counselors have traditionally educated patients and families about genetics, the representation of genetic counselors employed by the DTC GT industry as genetic educators for physicians appears to be changing as a result of the DTC GT product. A precursor to this may be the emphasis that earlier genetic testing companies, such as Myriad, placed on physician education. Myriad Genetics has been offering a commercial BRACAnalysis genetic susceptibility test for hereditary breast cancer since 1996 and is often seen as a forerunner to the large number of DTC GT companies now in operation (Matloff and Caplan 2008). Myriad invested heavily in patient and physician education, providing extensive online resources and supporting Continuing Medical Education programs for physicians (Williams-Jones and Graham 2003, p285). The training of doctors in genetics and genetic testing is a delicate issue for genetic counselors, considering their maintenance of professional identity and boundaries (Kenen 1997, p1381; Skirton et al. 2010).

### Product Advice

Closely related to their role in physician education is the genetic counselor’s role in providing product advice to healthcare providers and other consumers. The limited training that doctors receive in genetics (Powell et al. 2012) and the requirement for physicians to order the tests in some parts of the world at least partly explains why genetic counselors are represented as becoming involved in providing product advice to physicians. While 47 % of hypothetical users of DTC GT are confident that physicians have enough knowledge to interpret the test (McGuire et al. 2009, p7), research has shown that doctors in fact have very little knowledge of genetics, which leads some to claim that doctors are likely to “mishandle, misinterpret, and misadvise these patients on what is one of the most important pieces of medical information they will ever receive” (Matloff and Caplan 2008, p7). It is also important to keep in mind that due to various regulations, in a few states of the U.S. and in some countries in Europe, DTC GT must be ordered through a physician. Several companies are also moving towards a model whereby physicians must order the test (Howard and Borry 2011). The DTC GT industry is thus reliant upon doctors being knowledgeable of genetics and the genetic tests. Just as pharmaceutical representatives have an important role to play in educating physicians about new products (Martin 2006; Prosser and Walley 2006)—what Oldani (2004, p334) referred to as “the art of selling without selling”—genetic counselors working in the DTC GT industry may have a role in educating these potential consumers about genetic testing products. This raises a number of ethical questions in regards to conflicts of interest for genetic counselors employed by the DTC GT company.

### Lifestyle/Health Behavior Advice

The type of genetic information that is provided by DTC GT differs from the monogenetic information for which genetic counselors have traditionally been trained. This means that more traditional roles for genetic counselors, such as risk interpretation and prevention strategies, are, we argue, taking on new forms, as genetic counselors may be called upon to interpret an ambiguous product that provides information about a large number of genetic associations for complex diseases, based on controversial studies of specific populations, which provide probabilities that are very similar to population risk. One result of this shift, as we observed on the websites, and as O’Daniel (2010) has also noted, is that in dealing with DTC GT results, genetic counselors are represented as moving towards the provision of preventive health and lifestyle advice about complex disorders, based on small differences in risk.

Some genetic counselors writing in blogs are recognizing these roles are “non-traditional,” with a GeneDx counselor commenting that “having genetic counselors work outside their traditional roles, ensures we will have well informed professionals in these new areas of growth, that benefits not only doctors and counselors, but also patients and families” (Waltho 2011). Another counselor asks, in the same blog, “in a world … described as ‘woefully unprepared’ for the era of genomic medicine now approaching with all the subtlety and control of a locomotive off the tracks, the question lingers: where will we find ourselves, in this new landscape?” (Hercher 2009). Some of the roles that we have identified in this paper have also been recognized by other researchers, and could be considered activities that many genetic counselors engage in, in some form. For example, Wade and Wilfond (2006, p291) have gestured towards genetic counselors’ roles in educating physicians, nurses and health educators, whilst Zilliacus et al. (2010, p468) point out that genetic counselors also facilitate communication between the patient and clinicians. Hennen et al. (2010, p183) wonder whether genetic counselors working in the DTC GT industry are merely facilitating sales transactions. More generally, the “non-traditional” roles of genetic counselors working in the private sector and concerning communication technologies was also identified by Kenen (1997) in the late 1990s. While the roles we identified in our analysis may not be completely new roles for genetic counselors, they are shifting roles within the context of DTC GT, where what is novel is the setting in which such genetic counseling is occurring.

The emerging roles for genetic counselors represented on the websites that we have discussed are implicitly aligned with representations of more clinically acceptable, or “traditional” forms of genetic counseling on the websites. This occurs predominantly through the use of images that show face-to-face counseling sessions, with many counselors emphasizing their clinical experience in their online personal profiles. This is a crucial aspect of the genetic counseling product provided by DTC GT companies, as these more “traditional” images reinforce the test as a test with clinical significance, thus potentially strengthening consumer faith in the product. We need more research in this area to understand this further. DTC GT includes ambiguous products situated in a gray area between recreational/informational tests and medical tests. By providing genetic counseling services, DTC GT companies are drawing on associations of genetic counseling with clinical services and monogenetic testing. This image includes the professional attributes of autonomy, expertise, and non-directiveness.

In summary, our analysis demonstrates that direct-to-consumer genetic testing is leading to important and controversial changes in how the roles of genetic counselors are represented, as well as more broadly through: the utilization of telegenetics for a range of consumers, not only those who are specifically rurally or culturally isolated; the genetic counselors’ entrepreneurial involvement with commercial enterprise; and the new kinds of genetic information being provided by GWAS tests, differing from the monogenetic results that genetic counselors have traditionally dealt with. The participation of genetic counselors in the DTC GT industry raises ethical questions about professional boundaries and moral obligations, highlighting this as an area of professional concern.

The involvement of genetic counselors in the genetic testing industry also has a role to play in shaping the direct-to-consumer genetic testing product. Not only do genetic counselors potentially act as markers of clinical trust on the website, as discussed above, but the presence of the genetic counselor also raises interesting questions in regard to the “direct-ness” of genetic tests sold to the consumer, a common point of concern for those critical of DTC GT. As discussed earlier, much research about DTC GT considers “direct-to-consumer” to mean the selling of genetic tests to the public unmediated by a physician. Our analysis suggests that genetic counselors introduce a mediating aspect into the service, which has previously been underexplored in the literature. In dealing directly with physicians, either through education or product advice, genetic counselors working within the DTC GT industry also highlight that we need to consider carefully not only what we mean by “direct,” but also what we mean by “consumer.”

### Study Limitations

Our analysis of the online DTC GT landscape is limited by our focus on testing for mental disorders and by our use of English language search terms. Nonetheless the sample our search generated does seem to be representative of the range of websites available in this field, at least for websites registered in English-speaking parts of the world, where DTC GT is taking flight. Another limitation of the study is our focus on analyzing the online representations of genetic counseling, rather than empirically examining the actual work and practices performed by those employed in the DTC GT industry. More research is needed to investigate this area, including interviews with genetic counselors employed by DTC GT companies and participant-observer fieldwork. However, our research has shed light on an understudied aspect of DTC GT, and generated many questions upon which such research could be based, some of which we explore in the conclusion.

## Conclusions

Genetic counseling in the DTC GT industry is a controversial area of genomic medicine. DTC GT is resulting in the representation of emerging forms of expertise for genetic counselors. We find, as Kenen (1997, p1384) observed in the 1990s, that “genetic counselors find themselves once again actors in a sociomedical context fraught with ethical and social concerns compounded by changes in the way genetic services are delivered.” The literature is divided between those who argue that genetic counselors need to be involved with interpretation of DTC GT results, considering their expertise in risk communication and analysis (O’Daniel 2010, p322; Weaver and Pollin 2012, p365), and those who argue that DTC GT is outside their jurisdiction, and distracts from “the real work of answering the questions that counselors have been asked and giving information in which they have real confidence” (Clarke and Thirlaway 2011).

Rather than take sides in this debate, in this paper we have critically explored the roles that genetic counselors are represented to be assuming as part of the DTC GT industry. As mentioned, our research has raised many questions for further research. First, the normative aspect of genetic counseling and DTC GT needs closer examination. As Wade and Wilfond (2006, p289) write, “simply because a test is ‘genetic’ does not mean that a genetic counselor is always the clinician best suited to handling the case.” Other genetics experts may be more appropriate in this context, such as genetic nurses (Lea et al. 1998; Weaver and Pollin 2012), who may be more comfortable with giving lifestyle advice, and have experience with common health conditions. There is more to research empirically regarding the provision of genetic counseling through commercial internet sites. If researchers question the role of telegenetics for “more complex cases” (Zilliacus et al. 2010, p470), where does this leave the role of telegenetic counseling for complex disease information provided by DTC GT, especially for mental illness which arguably requires greater support (Zilliacus et al. 2010, p470)? If, as Boenink (2008, p60) writes, “the counseling trajectory can be conceptualized as a shared tinkering with divergent tools and means to fight a host of uncertainties,” how will genetic counselors in the DTC GT industry engage in tinkering through a single phone call or through internet interaction? How does the medium affect what information genetic counselors give, and how genetic counselors are accessed? How will genetic counselors themselves use the internet, or interactive features of the medium, during their counseling sessions?

In general, little is known about the genetic counselors working for these companies, and what they think about what DTC GT means for their profession and their professional identity. Is there any room left for uncertainty in their sessions and how are they dealing with an ambiguous product that absolves itself from diagnosis, yet is sold on the basis of providing health information? We also know little about how users are engaging with these genetic counseling services, although recent research has shown a low-uptake of the free genetic counseling offered by one of these sites (Bloss et al. 2011). In saying this, very little is also known about who uses DTC GT services more broadly, and there is still much to research about this emerging service. Similarly, in an industry with inter- and intra-national differences in regulatory mechanisms, there is more to consider in terms of the ways in which genetic counseling, as part of DTC GT, should be regulated.

How genetic counselors are involved in the DTC GT industry needs serious consideration. In this paper we have shown that genetic counselors are represented as assuming shifting roles, and promoting emerging forms of expertise in the DTC GT industry. The potential diversification of roles for genetic counselors will help them to maintain a particular position in the healthcare marketplace (Nancarrow and Borthwick 2005).We have discussed possible explanations for these emerging forms of counseling being represented, and placed our observations within a historical, social and economic context. Our analysis provides an important starting point for further research in this area, raising a number of policy issues. As genetic counselors, alongside physicians affiliated with the DTC GT companies, are increasingly becoming entangled with this commercial product, there is much more work to be done to understand what their involvement means for both the profession of genetic counseling, and the meaning of direct-to-consumer genetic testing.

## Footnotes

1 While in a ‘traditional’ genetic counseling session a carrier may be considered a patient, this is not necessarily the case in DTC GT.

## References

[CR1] Abrams D, Geier M (2006). A comparison of patient satisfaction with telehealth and on-site consultations: a pilot study for prenatal genetic counseling. Journal of Genetic Counseling.

[CR2] American Board of Genetic Counseling. (2009). Practice-based competencies. https://doi.org/www.abgc.net/docs/Practice%20Based%20Competencies_Aug%202006%2010-29-09.pdf. Accessed 6 January 2012.

[CR3] American College of Medical Genetics Board of Directors (2004). ACMG statement on direct-to-consumer genetic testing. Genetics in Medicine.

[CR4] American College of Obstetricians and Gynecologists (2008). ACOG committee opinion no. 409: Direct-to-consumer marketing of genetic testing. Obstetrics and Gynecology.

[CR5] Arribas-Ayllon, M., Sarangi, S., Clarke, A. (2012). Ethical decision-making in expert and family systems: The dynamics of trust-distrust in genetic testing. Paper presented on the 29th June at the Tenth Interdisciplinary Conference Communication, Medicine & Ethics, Trondheim, Norway.

[CR6] Bloss C, Schork N, Topol E (2011). Effect of direct-to-consumer genomewide profiling to assess disease risk. The New England Journal of Medicine.

[CR7] Boenink M, de Vries G, Horstman K (2008). Genetic diagnostics for hereditary breast cancer: displacement of uncertainty and responsibility. Genetics from laboratory to society.

[CR8] Borry P, Howard H (2008). DTC genetic services: a look across the pond. The American Journal of Bioethics.

[CR9] Clarke A, Thirlaway K (2011). ‘Genomic counseling’? Genetic counseling in the genomic era. Genome Medicine.

[CR10] Couzin J (2008). Gene tests for psychiatric risk polarize researchers. Science.

[CR11] de Vries G, de Vries G, Horstman K (2008). The ‘unknown’ practice of genetic testing. Genetics from laboratory to society.

[CR12] Denzin N, Lincoln Y (2011). The SAGE handbook of qualitative research.

[CR13] European Society of Human Genetics (2010). Statement of the ESHG on direct-to-consumer genetic testing for health-related purposes. European Journal of Human Genetics.

[CR14] Featherstone K, Atkinson P, Bharadwaj A, Clarke A (2006). Risky relations: family, kinship and the new genetics.

[CR15] Finucane B (2012). 2012 National society of genetic counselors presidential address: maintaining our professional identity in an ever-expanding genetics universe. Journal of Genetic Counseling.

[CR16] Genetics and Public Policy Center. (2006). *Direct-to-consumer genetic testing: empowering or endangering the public?* https://doi.org/www.dnapolicy.org/policy.issue.php?action=detail&issuebrief_id=32. Accessed 10 May 2011 (Archived by WebCite® at https://doi.org/www.webcitation.org/5znzdZmqv).

[CR17] Genetics and Public Policy Center. (2010). *Direct-to-consumer genetic testing companies*. https://doi.org/www.dnapolicy.org/resources/AlphabetizedDTCGeneticTestingCompanies.pdf. Accessed 10 May 2011 (Archived by WebCite® at https://doi.org/www.webcitation.org/5znzI162L).

[CR18] Genetics and Public Policy Center. (2011). *Direct-to-consumer genetic testing companies*. https://doi.org/www.dnapolicy.org/news.release.php?action=detail&pressrelease_id=145. Accessed 17 August 2011 (Archived by WebCite® at https://doi.org/www.webcitation.org/610DDbCns).

[CR19] Guttmacher AE, Collins FS (2003). Welcome to the genomic era. The New England Journal of Medicine.

[CR20] Hall S (1997). Representation: cultural representation and signifying practices.

[CR21] Hawkins A, Ho A (2012). Genetic counseling and the ethical issues around direct to consumer genetic testing. Journal of Genetic Counseling.

[CR22] Hennen L, Sauter A, Van Den Cruyce E (2010). Direct to consumer genetic testing: insights from an internet scan. New Genetics and Society.

[CR23] Hercher, L. (2009). *DC takes on DTC: The “T” doesn’t stand for tomorrow anymore*. https://doi.org/thednaexchange.com/2009/09/04/dc-takes-on-dtc-the-t-doesnt-stand-for-tomorrow-anymore. Accessed 28 April (Archived by WebCite® at https://doi.org/www.webcitation.org/5yHgvXiTK).

[CR24] Hock K, Christensen K, Yashar B, Roberts JS, Gollust SE, Uhlmann W (2011). Direct-to-consumer genetic testing: an assessment of genetic counselors’ knowledge and beliefs. Genetics in Medicine.

[CR25] Howard Heidi Carmen, Borry Pascal (2011). Is there a doctor in the house?. Journal of Community Genetics.

[CR26] Hudson K, Javitt G, Burke W, Byers P, Social Issues ASHG, ASHG Social Issues Committee (2007). ASHG statement on direct-to-consumer genetic testing in the United States. The American Journal of Human Genetics.

[CR27] Jordens CFC, Kerridge IH, Samuel GN (2009). Direct-to-consumer personal genome testing: the problem is not ignorance it is market failure. The American Journal of Bioethics.

[CR28] Kenen RH (1997). Opportunities and impediments for a consolidating and expanding profession: genetic counseling in the United States. Social Science & Medicine.

[CR29] Koch L, Nordahl Svendsen M (2005). Providing solutions-defining problems: the imperative of disease prevention in genetic counselling. Social Science & Medicine.

[CR30] Lea DH, Jenkins JF, Francomano CA (1998). Genetics in clinical practice: new directions for nursing and health care.

[CR31] Leighton JW, Valverde K, Bernhardt BA (2012). The general public’s understanding and perception of direct-to-consumer genetic test results. Public Health Genomics.

[CR32] Lupton D, Petersen A, Bunton R (1997). Foucault and the medicalisation critique. Foucault, health and medicine.

[CR33] Martin E (2006). Pharmaceutical virtue. Culture, Medicine and Psychiatry.

[CR34] Matloff E, Caplan A (2008). Direct to confusion: lessons learned from marketing BRCA testing. The American Journal of Bioethics.

[CR35] McGowan ML, Fishman JR, Lambrix MA (2010). Personal genomics and individual identities: motivations and moral imperatives of early users. New Genetics and Society.

[CR36] McGuire AL, Diaz CM, Wang T, Hilsenbeck SG (2009). Social networkers’ attitudes toward direct-to-consumer personal genome testing. The American Journal of Bioethics.

[CR37] Nancarrow SA, Borthwick AM (2005). Dynamic professional boundaries in the healthcare workforce. Sociology of Health & Illness.

[CR38] National Society of Genetic Counselors. (2010a). *Consumers should be mindful of DTC genetic testing: Don’t bypass genetic counselors, medical geneticists or other healthcare providers*. Chicago. https://doi.org/www.nsgc.org/client_files/news/040609dtc.pdf. Accessed 5 May 2011 (Archived by WebCite® at https://doi.org/www.webcitation.org/5zx2YwWhX).

[CR39] National Society of Genetic Counselors. (2010b). *National Society of Genetic Counselors Professional Status Survey 2010, Executive Summary*. https://doi.org/www.nsgc.org/Publications/ProfessionalStatusSurvey/tabid/142/Default.aspx. Accessed 6 January 2012.

[CR40] National Society of Genetic Counselors. (2011). *FAQs about Genetic Counselors and the NSGC*. https://doi.org/www.nsgc.org/About/FAQsaboutGeneticCounselorsandtheNSGC/tabid/143/Default.aspx. Accessed 11 August (Archived by WebCite® at https://doi.org/www.webcitation.org/60qxjfXLH).

[CR41] Nordgren A, Juengst ET (2009). Can genomics tell me who I am? Essentialistic rhetoric in direct-to-consumer DNA testing. New Genetics and Society.

[CR42] Novas C, Rose N (2000). Genetic risk and the birth of the somatic individual. Economy and Society.

[CR43] Nuffield Council on Bioethics. (2010). *Medical profiling and online medicine: The ethics of ‘personalised healthcare’ in a consumer age*. London: Nuffield Council on Bioethics. https://doi.org/www.nuffieldbioethics.org/personalised-healthcare-0. Accessed 20 February 2011 (Archived by WebCite® at https://doi.org/www.webcitation.org/5zx5028ky).

[CR44] O’Daniel J (2010). The prospect of genome-guided preventive medicine: a need and opportunity for genetic counselors. Journal of Genetic Counseling.

[CR45] Oldani MJ (2004). Thick prescriptions: toward an interpretation of pharmaceutical sales practices. Medical Anthropology Quarterly.

[CR46] Pagon RA (2002). Genetic testing for disease susceptibilities: consequences for genetic counseling. Trends in Molecular Medicine.

[CR47] Parthasarathy S (2010). Assessing the social impact of direct-to-consumer genetic testing: understanding sociotechnical architectures. Genetics in Medicine.

[CR48] Petrakaki D, Cornford T, Hibberd R, Lichtner V, Barber N (2011). The role of technology in shaping the professional future of community pharmacists: the case of the electronic prescription service in the English National Health Service. Researching the Future in Information Systems.

[CR49] Powell K, Christianson C, Cogswell W, Dave G, Verma A, Eubanks S, Henrich V (2012). Educational needs of primary care physicians regarding direct-to-consumer genetic testing. Journal of Genetic Counseling.

[CR50] Prosser H, Walley T (2006). New drug prescribing by hospital doctors: the nature and meaning of knowledge. Social Science & Medicine.

[CR51] Rees G, Young M-A, Gaff C, Martin P (2006). A qualitative study of health professionals’ views regarding provision of information about health-protective behaviors during genetic consultation for breast cancer. Journal of Genetic Counseling.

[CR52] Richards M (2010). Reading the runes of my genome: a personal exploration of retail genetics. New Genetics and Society.

[CR53] Skirton H, Lewis C, Kent A, Coviello DA (2010). Genetic medicine and the challenge of genomic medicine: development of core competencies to support preparation of health professionals in Europe. European Journal of Human Genetics.

[CR54] Stalker HJ, Wilson R, McCune H, Gonzalez J, Moffett M, Zori RT (2006). Telegenetic medicine: improved access to services in an underserved area. Journal of Telemedicine and Telecare.

[CR55] Udesky L (2010). The ethics of direct-to-consumer genetic testing. The Lancet.

[CR56] Wade CH, Wilfond BS (2006). Ethical and clinical practice considerations for genetic counselors related to direct-to-consumer marketing of genetic tests. American Journal of Medical Genetics. Part C, Seminars in Medical Genetics.

[CR57] Waltho, S. (2011). *Learning to create opportunity*. https://doi.org/thednaexchange.com/2011/03/17/learning-to-create-opportunity. Accessed 21 April 2011 (Archived by WebCite® at https://doi.org/www.webcitation.org/5y6bmbwtT).

[CR58] Weaver M, Pollin T (2012). Direct-to-consumer genetic testing: what are we talking about?. Journal of Genetic Counseling.

[CR59] Williams-Jones B, Graham JE (2003). Actor-network theory: a tool to support ethical analysis of commercial genetic testing. New Genetics and Society.

[CR60] Zilliacus E, Meiser B, Lobb E, Kirk J, Warwick L, Tucker K (2010). Women’s experience of telehealth cancer genetic counseling. Journal of Genetic Counseling.

